# Supplementary material for: AmpliPhy improves gene trees by adding homologous sequences without affecting alignments
Source: Bioinform Adv. 2026 Aug 7;6(1):vbag222. doi: 10.1093/bioadv/vbag222 (PMC13516908; doi:10.1093/bioadv/vbag222)
Supplement: Supplementary file 1 [file vbag222_supplementary_data.pdf]

AmpliPhy improves gene trees by adding homologous sequences without affecting alignments

Dongwook Kim [1,2,*], Manuel Gil [2,3], Kazutaka Katoh [4,5] and Christophe Dessimoz [1,2]

[1]Department of Computational Biology, University of Lausanne, Lausanne, Switzerland, [2]Swiss Institute of Bioinformatics, Lausanne, Switzerland, [3]Institute of Computational Life Science, Zürich University of Applied Sciences, Wädenswil, Switzerland, [4]Department of Genome Informatics, Research Institute for Microbial Diseases, Osaka University, Suita, Japan, [5]Department of Computational Biology and Medical Sciences, Graduate School of Frontier Sciences, University of Tokyo, Kashiwa, Japan

SUPPLEMENTARY MATERIALS

Figures

- Supplementary Fig. 1. Effect of homolog enrichment on alignment and tree inference in Primates gene families.
- Supplementary Fig. 2. Effect of homolog enrichment on alignment and tree inference in large Eukaryotic gene families.
- Supplementary Fig. 3. Comparison of enrichment effect across different evolutionary models.
- Supplementary Fig. 4. Effect of homolog enrichment on alignment and tree inference in bacterial gene families.
- Supplementary Fig. 5. Reduced resolution of taxonomic congruence when applying NCBI Taxonomy to bacterial species.
- Supplementary Fig. 6. Decomposed effect of enrichment by the stratification of homologs.
- Supplementary Fig. 7. SPFN differences between impoverished and original MSAs against simulated ground-truth.
- Supplementary Fig. 8. Iterative tree builders outperform faster heuristic alternatives.
- Supplementary Fig. 9. Tree inference remains improving after the enrichment even after removing the rooting dependency.

Tables

- Supplementary Table 1. Comprehensive list of OMA root HOGs used in this study.
- Supplementary Table 2. List of aligners and command lines used to run the benchmark.
- Supplementary Table 3. Evolutionary model selected before and after enriching Amniota HOGs.
- Supplementary Table 4. Taxonomic impact on the gene trees by enrichment of Eukaryota HOGs.

*To whom correspondence should be addressed. Email: dongwook.kim@unil.ch

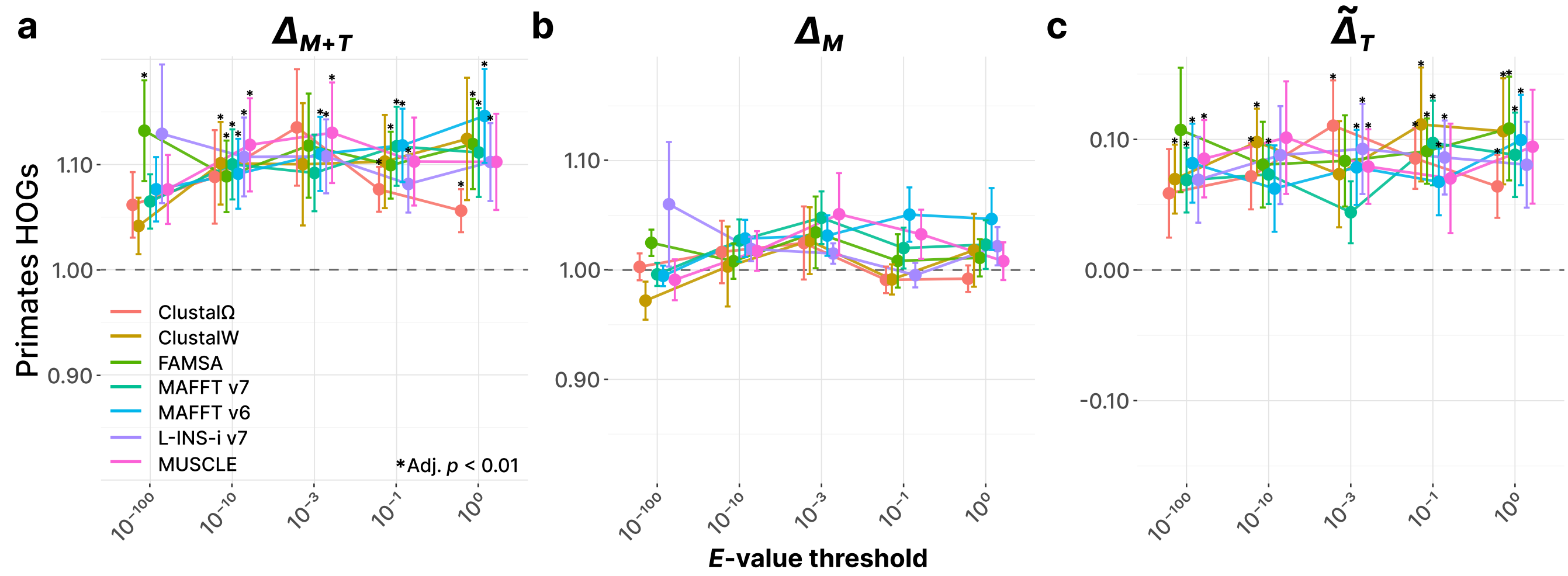

Supplementary Fig. 1. Effect of homolog enrichment on alignment and tree inference in Primates gene families. a. Combined effect on alignment and tree inference (Δ_{M+T}) after enriching 100 HOGs from the order Primates, measured by taxonomic congruence. **b.** Effect on alignment quality (Δ_M). **c.** Isolated effect on tree inference quality ($\tilde{\Delta}_T$). Statistically significant points (BH-adjusted $p < 0.01$) are annotated with asterisks.

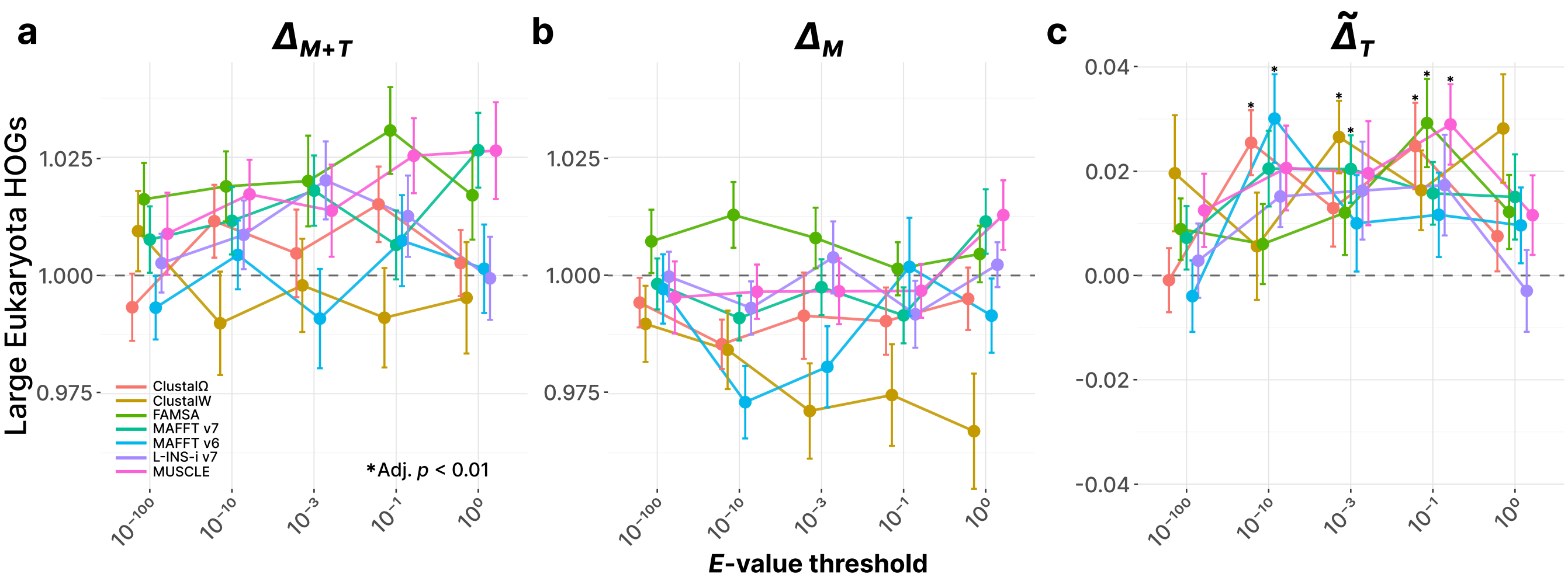

Supplementary Fig. 2. Effect of homolog enrichment on alignment and tree inference in large Eukaryotic gene families. a. Combined effect on alignment and tree inference (Δ_{M+T}) after enriching 100 HOGs from Eukaryotes with more than 100 member sequences. **b.** Effect on alignment quality (Δ_M). **c.** Isolated effect on tree inference quality ($\tilde{\Delta}_T$). Statistically significant points (BH-adjusted $p < 0.01$) are annotated with asterisks.

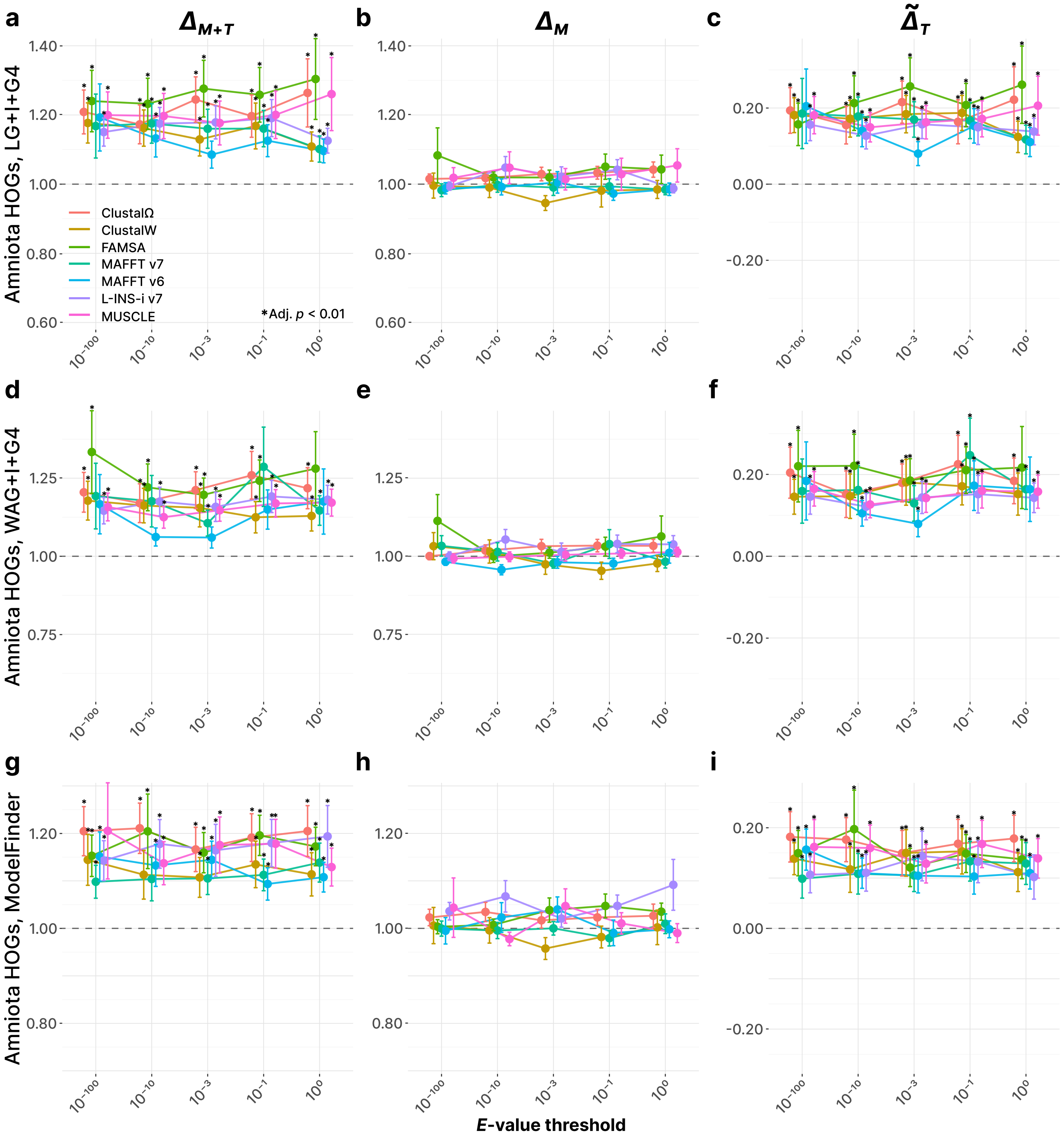

Supplementary Fig. 3. Comparison of enrichment effect across different evolutionary models. a. Combined effect on alignment and tree inference (Δ_{M+T}) after enriching 100 HOGs from the clade Amniota. Phylogenetic trees were inferred under the model `LG+I+G4`. **b.** Effect on alignment quality (Δ_M). **c.** Isolated effect on tree inference quality ($\tilde{\Delta}_T$). Statistically significant points (BH-adjusted $p < 0.01$) are annotated with asterisks. **d–f.** Corresponding data under evolutionary model `WAG+I+G4`. **g–i.** Corresponding data under the optimal model chosen by IQ-TREE 2 ModelFinder (`MF`).

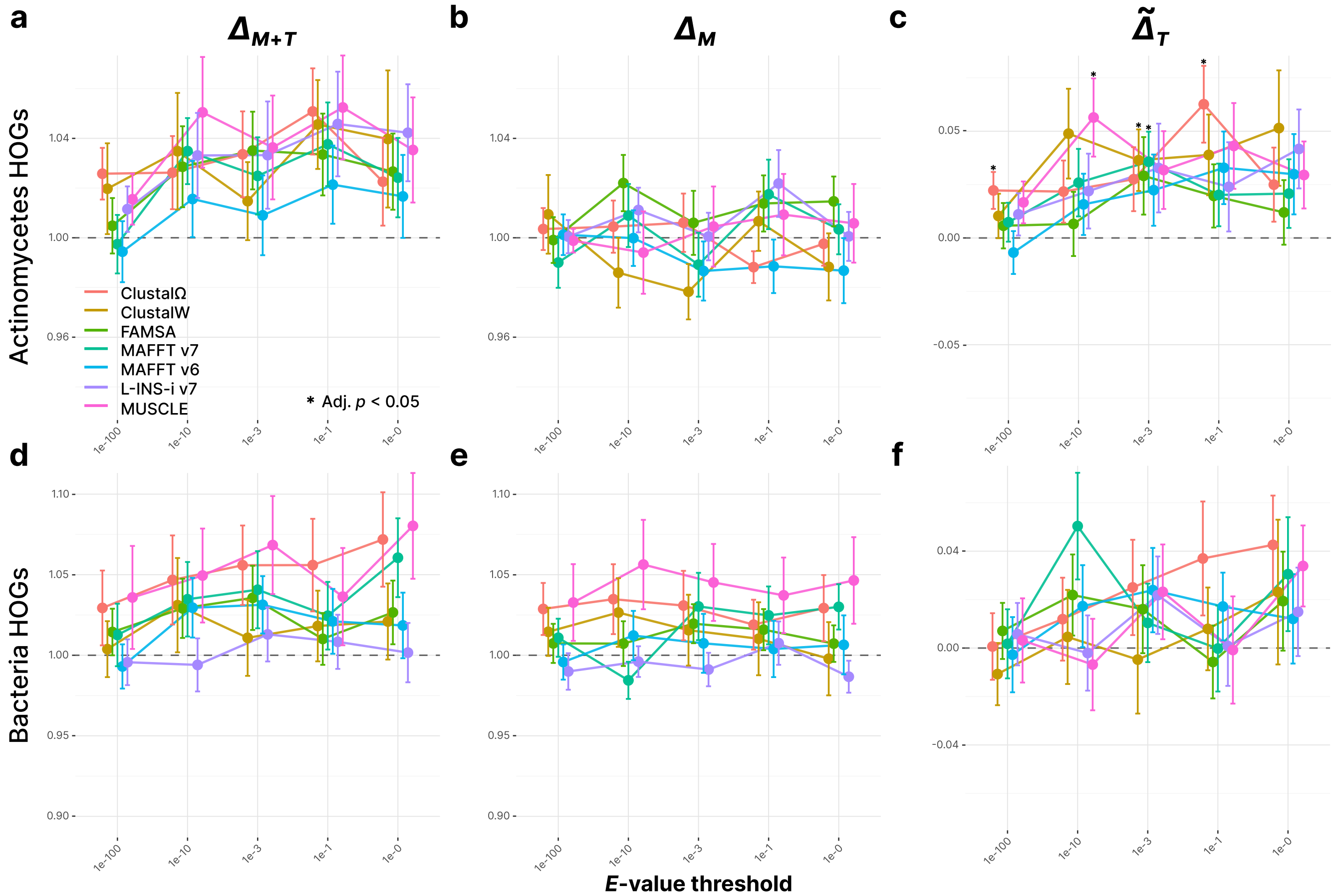

Supplementary Fig. 4. Effect of homolog enrichment on alignment and tree inference in bacterial gene families. a. Combined effect on alignment and tree inference (Δ_{M+T}) after enriching 100 HOGs from the bacterial class Actinomycetes, measured by taxonomic congruence. **b.** Effect on alignment quality (Δ_M). **c.** Isolated effect on tree inference quality ($\tilde{\Delta}_T$). Statistically significant points (BH-adjusted $p < 0.05$) are annotated with asterisks. **d–f.** Corresponding taxonomic congruence statistics for 100 HOGs spanning the entire bacterial domain.

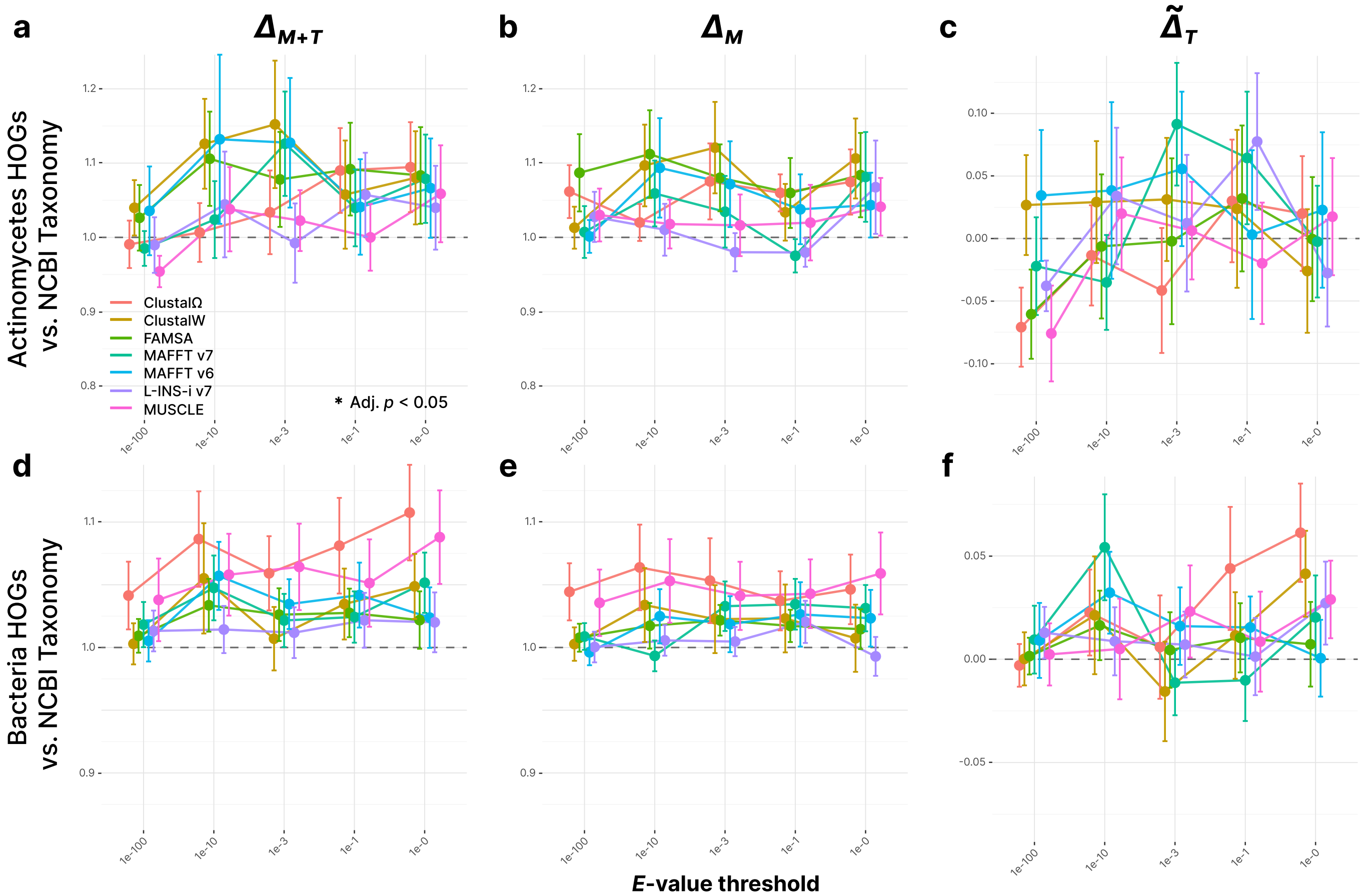

Supplementary Fig. 5. Reduced resolution of taxonomic congruence when applying NCBI Taxonomy to bacterial species. a. Combined effect on alignment and tree inference (Δ_{M+T}) for homolog-enriched Actinomycetes HOGs, where taxonomic congruence is computed using NCBI taxonomy instead of GTDB taxonomy. **b.** Effect on alignment quality (Δ_M). **c.** Isolated effect on tree inference quality ($\tilde{\Delta}_T$). **d–f.** NCBI-based taxonomic congruence statistics for 100 bacterial HOGs.

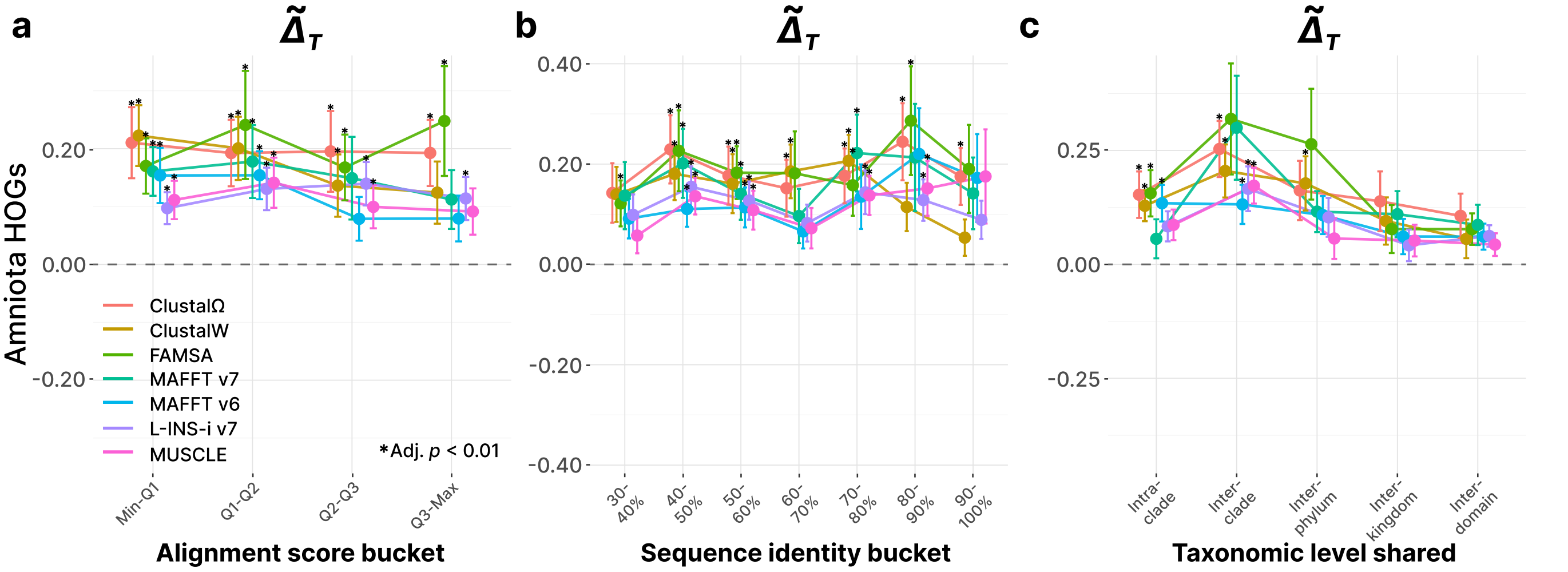

Supplementary Fig. 6. Decomposed effect of enrichment by the stratification of homologs. a. Impact of enrichment on the Aminota HOG tree inference quality ($\tilde{\Delta}_T$), with the homologs divided into 4 identical sized buckets based on their alignment bit scores. **b.** Impact on the tree inference with homologs grouped based on their sequence identity with respect to the query sequence ($fident$, see Methods for the definition). **c.** Impact on the tree inference with homologs grouped by their taxonomic relationship with the query clade (Amniota). Statistically significant points (BH-adjusted $p < 0.01$) are annotated with asterisks.

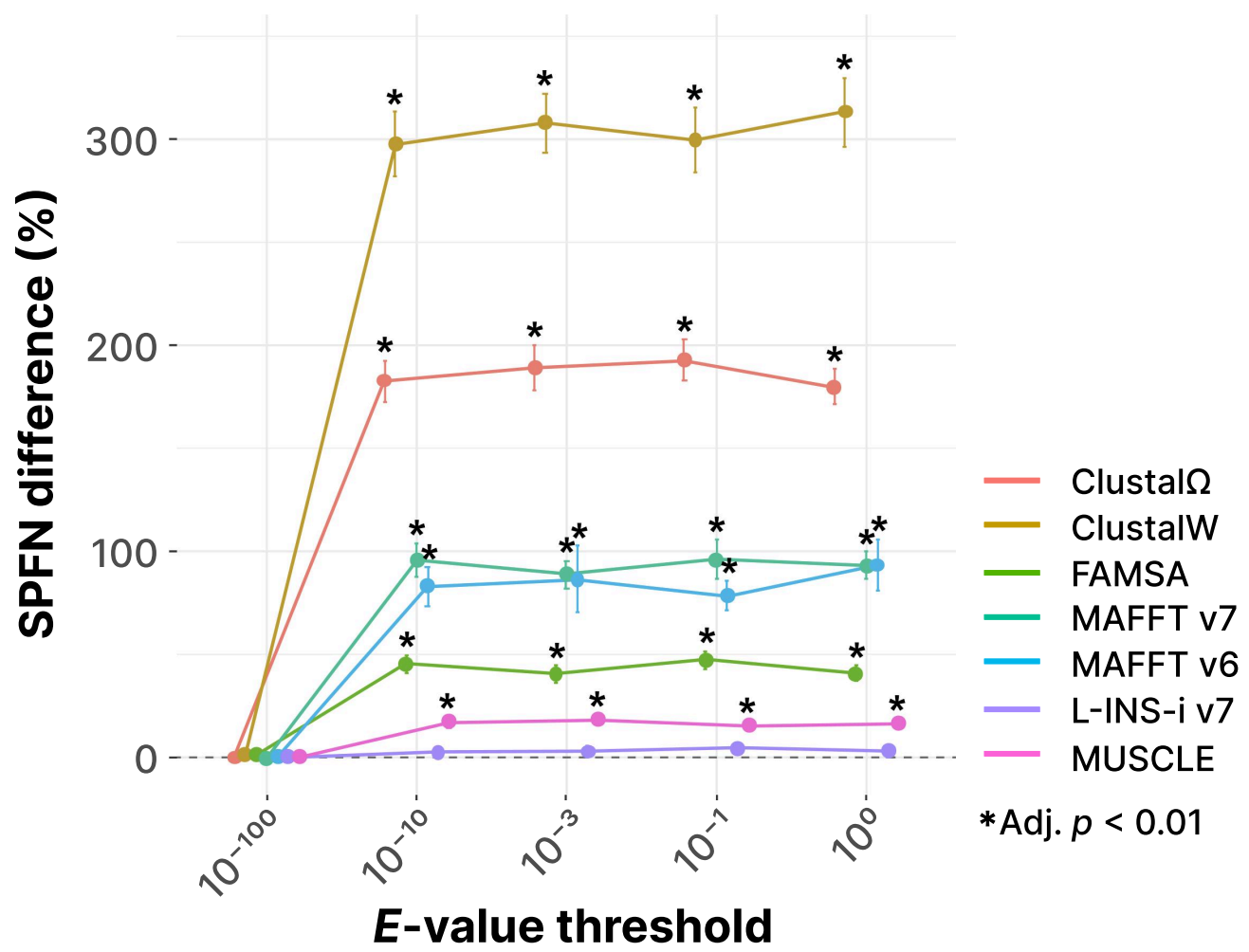

Supplementary Fig. 7. SPFN differences between impoverished and original MSAs against simulated ground-truth. Relative difference of SPFN values of impoverished MSAs against original MSAs, compared to ground-truth alignment. Statistically significant points (BH-adjusted $p < 0.01$) are annotated with asterisks.

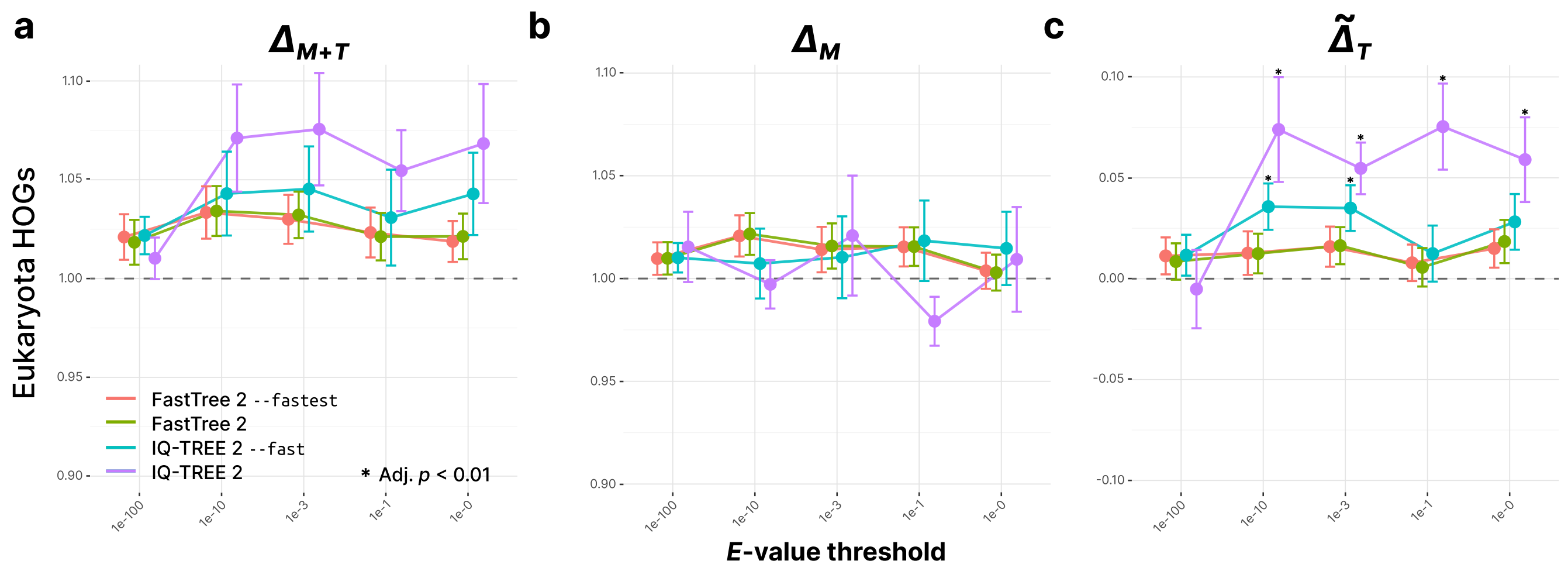

Supplementary Fig. 8. Iterative tree builders outperform faster heuristic alternatives. Data points are colored by the tree-building program used for phylogenetic inference, while the sequence aligner was fixed to MAFFT. **a.** Combined effect of sequence addition on alignment and tree inference (Δ_{M+T}) for 100 Eukaryota HOGs. **b.** Effect on alignment quality (Δ_M). **c.** Difference between Δ_{M+T} and Δ_M ($\tilde{\Delta}_T$; effect on tree inference quality). Statistically significant points (BH-adjusted $p < 0.01$) are annotated with asterisks.

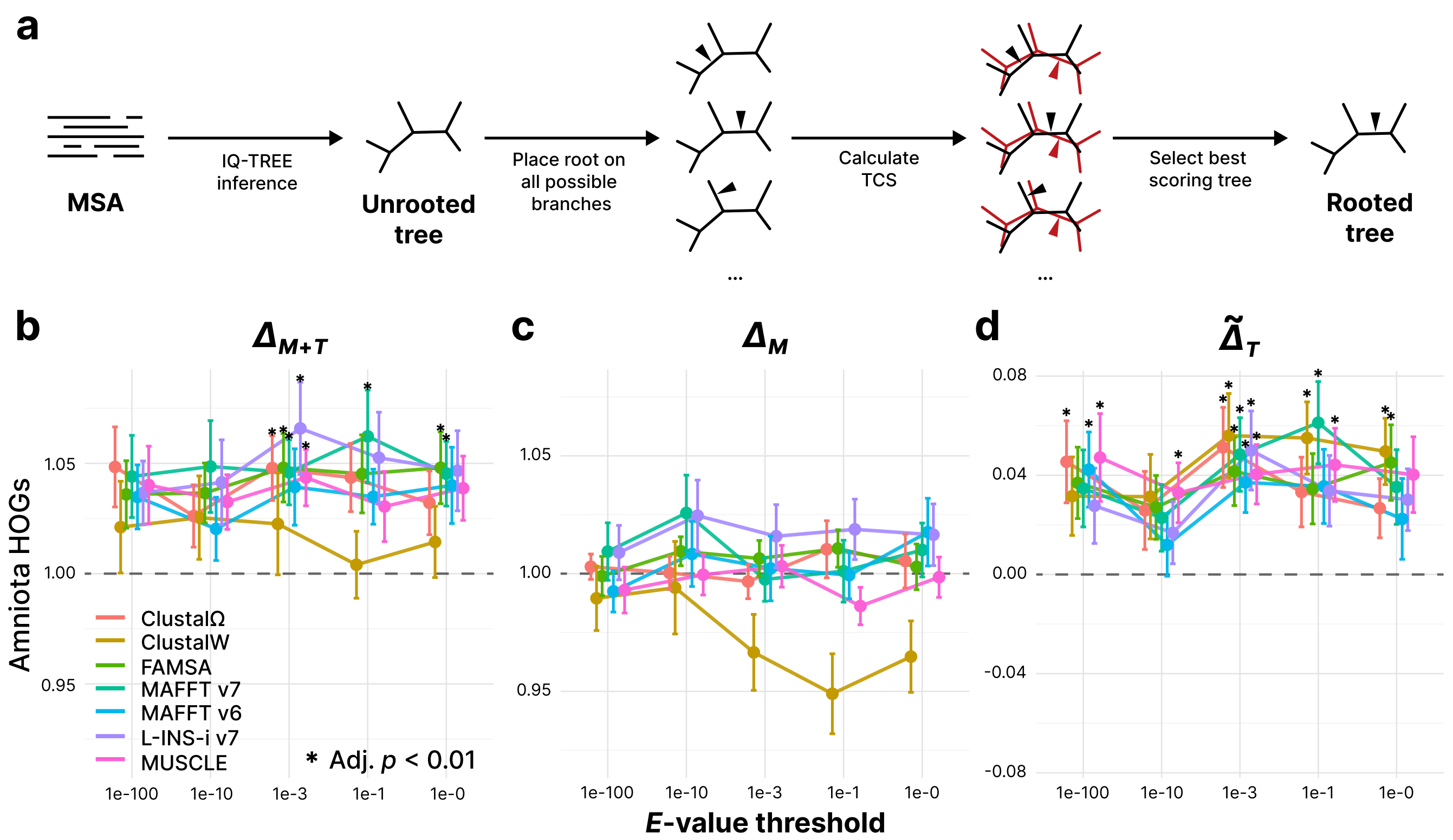

Supplementary Fig. 9. Tree inference remains improving after the enrichment even after removing the rooting dependency. a. Schematic illustration of the removal of rooting dependency of TCS. From the inferred unrooted tree, we calculated taxonomic congruence scores from the set of rooted trees obtained by placing root on all possible branches. Among them, we selected the rooted tree with the highest score. **b.** Combined effect on alignment and tree inference (Δ_{M+T}) after enriching 100 Amniota HOGs with removed rooting dependency. **c.** Effect on alignment quality (Δ_M). **d.** Isolated effect on tree inference quality ($\tilde{\Delta}_T$). Statistically significant points (BH-adjusted $p < 0.01$) are annotated with asterisks.

Supplementary Table 1. Comprehensive list of OMA root HOGs used in this study. Each HOG ID can be accessed in the OMA browser by querying `HOG:<HOG ID>`. The benchmark set indicates the taxon set in which each HOG was used. Taxonomic level indicates the taxon at which each HOG was inferred. Functional descriptions were retrieved from the OMA database.

HOG ID	Benchmark set	Taxonomic level	Functional description
E0736661	Eukaryota	Tetrapoda	Leukotriene B4 receptor
E0736928	Eukaryota	Tetrapoda	KAT8 regulatory NSL complex subunit
E0737227	Eukaryota	Tetrapoda	CD6 molecule
E0737958	Eukaryota	Sarcopterygii	ATPase H+ transporting accessory protein
E0738913	Eukaryota	Euteleostomi	NK6 homeobox
E0738995	Eukaryota	Euteleostomi	BMERB domain containing
E0739069	Eukaryota	Euteleostomi	HAUS augmin like complex subunit
E0739275	Eukaryota	Euteleostomi	Mitogen-activated protein kinase kinase kinase 12
E0739309	Eukaryota	Euteleostomi	Myeloid-associated differentiation marker
E0739422	Eukaryota	Euteleostomi	Sema domain immunoglobulin domain Ig short basic domain secreted semaphorin 3E
E0739485	Eukaryota	Euteleostomi	High mobility group nucleosome-binding domain-containing protein
E0739504	Eukaryota	Euteleostomi	Hydroxysteroid 17-beta dehydrogenase
E0739554	Eukaryota	Euteleostomi	Angiopoietin like
E0739875	Eukaryota	Euteleostomi	Macrophage scavenger receptor
E0741827	Eukaryota	Euteleostomi	F-box protein 24
E0745703	Eukaryota	Gnathostomata	Matrix metalloproteinase-9
E0745717	Eukaryota	Gnathostomata	Protein-coupled receptor 20
E0745736	Eukaryota	Gnathostomata	Prostaglandin F2-alpha receptor
E0745773	Eukaryota	Gnathostomata	Tumor protein p53 inducible nuclear protein
E0745867	Eukaryota	Gnathostomata	Nucleotide binding oligomerization domain containing
E0745959	Eukaryota	Gnathostomata	Domain containing
E0745969	Eukaryota	Gnathostomata	Myozenin
E0746267	Eukaryota	Gnathostomata	Forkhead box N2
E0746286	Eukaryota	Gnathostomata	EvC ciliary complex subunit
E0746357	Eukaryota	Gnathostomata	Thromboxane A2 receptor
E0746423	Eukaryota	Gnathostomata	Hyaluronan binding protein
E0746523	Eukaryota	Gnathostomata	Family with sequence similarity 222 member
E0746543	Eukaryota	Gnathostomata	Rho guanine nucleotide exchange factor
E0746659	Eukaryota	Gnathostomata	Fibronectin type III domain containing
E0746710	Eukaryota	Gnathostomata	Nuclear receptor interacting protein
E0747054	Eukaryota	Gnathostomata	Six family member
E0747393	Eukaryota	Gnathostomata	KAT8 regulatory NSL complex subunit
E0748047	Eukaryota	Gnathostomata	Macrophage receptor with collagenous structure
E0748254	Eukaryota	Gnathostomata	TAFA chemokine like family member
E0749133	Eukaryota	Gnathostomata	DnaJ heat shock protein family Hsp40 member B2
E0749136	Eukaryota	Gnathostomata	Claudin
E0749142	Eukaryota	Gnathostomata	Poly ADP-ribose polymerase
E0749861	Eukaryota	Gnathostomata	Origin recognition complex subunit
E0752475	Eukaryota	Vertebrata	5-hydroxytryptamine receptor 3A
E0752720	Eukaryota	Vertebrata	Fidgetin microtubule severing factor
E0753547	Eukaryota	Vertebrata	Tripartite motif containing 55
E0754197	Eukaryota	Vertebrata	Lysosomal associated membrane protein
E0754233	Eukaryota	Vertebrata	CACN subunit beta associated regulatory protein
E0756359	Eukaryota	Chordata	ETS variant transcription factor
E0758308	Eukaryota	Chordata	Gap junction protein
E0758684	Eukaryota	Chordata	Collagen type IX alpha
E0759084	Eukaryota	Deuterostomia	G-protein coupled receptor
E0759259	Eukaryota	Deuterostomia	Peroxisome proliferator-activated receptor
E0759639	Eukaryota	Deuterostomia	Family with sequence similarity 126 member
E0761639	Eukaryota	Bilateria	Midnolin
E0769246	Eukaryota	Bilateria	Doublecortin domain containing
E0770269	Eukaryota	Bilateria	Transcription factor
E0778401	Eukaryota	Eumetazoa	E3 ubiquitin-protein ligase
E0779894	Eukaryota	Eumetazoa	Subcomponent-binding protein mitochondrial
E0780058	Eukaryota	Eumetazoa	E2F transcription factor
E0780419	Eukaryota	Eumetazoa	Partner transcriptional co-repressor
E0781413	Eukaryota	Eumetazoa	Protein lin-52 homolog
E0781675	Eukaryota	Eumetazoa	Derived by automated computational analysis using gene prediction method Gnomon
E0781703	Eukaryota	Eumetazoa	Replication protein
E0781792	Eukaryota	Eumetazoa	Protein 10

Supplementary Table 1. (Continued.)

HOG ID	Benchmark set	Taxonomic level	Functional description
E0783173	Eukaryota	Eumetazoa	Plexin domain containing
E0784066	Eukaryota	Eumetazoa	MSL complex subunit
E0784079	Eukaryota	Eumetazoa	Cell division cycle associated
E0785287	Eukaryota	Eumetazoa	Trefoil factor
E0789185	Eukaryota	Metazoa	Cilia and flagella associated protein 276
E0789260	Eukaryota	Metazoa	Coiled-coil domain containing 186
E0790046	Eukaryota	Metazoa	Transmembrane protein 229b
E0790087	Eukaryota	Metazoa	E3 ubiquitin-protein ligase KCMF1
E0790149	Eukaryota	Metazoa	Nuclear factor of kappa light polypeptide gene enhancer in B-cells inhibitor epsilon
E0792964	Eukaryota	Opisthokonta	Exosome complex component rrp45
E0793021	Eukaryota	Opisthokonta	Spindle apparatus coiled-coil protein
E0793757	Eukaryota	Opisthokonta	Zinc finger hit domain-containing protein
E0793921	Eukaryota	Opisthokonta	Derived by automated computational analysis using gene prediction method Gnomon
E0794199	Eukaryota	Opisthokonta	Protein MIX23
E0794232	Eukaryota	Opisthokonta	Serine/threonine-protein kinase ulk3
E0794379	Eukaryota	Opisthokonta	Ankyrin repeat domain 16
E0795316	Eukaryota	Opisthokonta	Cox assembly mitochondrial protein
E0795420	Eukaryota	Opisthokonta	SH2 domain containing
E0796129	Eukaryota	Opisthokonta	Striatin interacting protein
E0801614	Eukaryota	Eukaryota	Eukaryotic translation initiation factor 2d
E0801674	Eukaryota	Eukaryota	Pescadillo homolog
E0801829	Eukaryota	Eukaryota	RRNA adenine
E0802240	Eukaryota	Eukaryota	Derived by automated computational analysis using gene prediction method Gnomon
E0802765	Eukaryota	Eukaryota	Saccharopine dehydrogenase NADP binding domain-containing protein
E0803072	Eukaryota	Eukaryota	Transmembrane protein 144
E0803107	Eukaryota	Eukaryota	Domain-containing protein
E0803212	Eukaryota	Eukaryota	Thioredoxin-related transmembrane protein
E0803328	Eukaryota	Eukaryota	Non-structural maintenance of chromosomes element
E0803575	Eukaryota	Eukaryota	Actin related protein 2/3 complex inhibitor
E0803937	Eukaryota	Eukaryota	SNARE-complex protein Syntaxin-18 N-terminal domain-containing protein
E0804722	Eukaryota	Eukaryota	Coiled-coil domain containing 113
E0804798	Eukaryota	Eukaryota	DNA replication licensing factor mcm2
E0804898	Eukaryota	Eukaryota	Cilia- and flagella-associated protein 300
E0805604	Eukaryota	Eukaryota	GPI ethanolamine phosphate transferase
E0808137	Eukaryota	Eukaryota	Guanine nucleotide exchange factor
E0812781	Eukaryota	Eukaryota	Membrane protein
E0813567	Eukaryota	Eukaryota	6-pyruvoyl tetrahydrobiopterin synthase
E0816287	Eukaryota	Eukaryota	DDB1 and CUL4 associated factor
E0816677	Eukaryota	Eukaryota	Alpha subcomplex subunit 10 mitochondrial
E0818004	Eukaryota	Eukaryota	TBC1 domain family member 23
E0732656	Amniota	Amniota	A-kinase anchor inhibitor
E0736903	Amniota	Tetrapoda	Receptor subunit alpha
E0738993	Amniota	Euteleostomi	Protein disulfide isomerase like testis expressed
E0739117	Amniota	Euteleostomi	Phosphoinositide-3-kinase interacting protein
E0739356	Amniota	Euteleostomi	Synapse defective Rho GTPase homolog
E0739363	Amniota	Euteleostomi	Potassium channel tetramerization domain containing
E0740076	Amniota	Euteleostomi	Nuclear receptor subfamily
E0740208	Amniota	Euteleostomi	Myelin protein zero like
E0745588	Amniota	Gnathostomata	Opsin
E0745662	Amniota	Gnathostomata	Complexin
E0745759	Amniota	Gnathostomata	Recombination-activating protein
E0746040	Amniota	Gnathostomata	Exocyst complex component
E0746607	Amniota	Gnathostomata	Dendrocyte expressed seven transmembrane protein
E0746839	Amniota	Gnathostomata	Matrix metallopeptidase 23B
E0747039	Amniota	Gnathostomata	C-X-C motif chemokine receptor
E0747049	Amniota	Gnathostomata	Proopiomelanocortin
E0748381	Amniota	Gnathostomata	Apolipoprotein A-IV
E0749003	Amniota	Gnathostomata	Ataxin
E0749052	Amniota	Gnathostomata	SIX homeobox
E0750288	Amniota	Gnathostomata	Interleukin 17 receptor
E0752398	Amniota	Vertebrata	Glucose-6-phosphatase catalytic subunit

Supplementary Table 1. (Continued.)

HOG ID	Benchmark set	Taxonomic level	Functional description
E0752660	Amniota	Vertebrata	Zinc finger and BTB domain containing 7a
E0752678	Amniota	Vertebrata	LIM and cysteine rich domains
E0754068	Amniota	Vertebrata	BARX homeobox
E0754499	Amniota	Vertebrata	Acetylcholine receptor subunit gamma
E0754546	Amniota	Vertebrata	Paired related homeobox
E0755467	Amniota	Chordata	EBF transcription factor
E0755569	Amniota	Chordata	Tyrosine-protein kinase receptor
E0755637	Amniota	Chordata	L-dopachrome tautomerase
E0756159	Amniota	Chordata	Dopamine receptor
E0756270	Amniota	Chordata	Retinol binding protein
E0756421	Amniota	Chordata	Glycerophosphodiester phosphodiesterase domain containing
E0759640	Amniota	Deuterostomia	ADAMTS like
E0760031	Amniota	Deuterostomia	SOSS complex subunit
E0761363	Amniota	Bilateria	Mediator of RNA polymerase ii transcription subunit 30
E0761506	Amniota	Bilateria	Secreted protein acidic and cysteine rich
E0761586	Amniota	Bilateria	Open reading frame 120
E0762353	Amniota	Bilateria	5-hydroxytryptamine receptor
E0771775	Amniota	Bilateria	Suppressor of variegation 3-9 homolog
E0778191	Amniota	Eumetazoa	ELL associated factor
E0778742	Amniota	Eumetazoa	Catenin
E0779849	Amniota	Eumetazoa	Serine/threonine-protein kinase 40
E0779919	Amniota	Eumetazoa	Leucine rich repeat containing 73
E0780141	Amniota	Eumetazoa	Islet cell autoantigen
E0780552	Amniota	Eumetazoa	Histone acetyltransferase
E0781053	Amniota	Eumetazoa	Synaptotagmin
E0781454	Amniota	Eumetazoa	Mucosa-associated lymphoid tissue lymphoma translocation protein
E0783327	Amniota	Eumetazoa	Zinc finger FYVE domain containing 27
E0784079	Amniota	Eumetazoa	Cell division cycle associated
E0789041	Amniota	Metazoa	Transcriptional adapter
E0789139	Amniota	Metazoa	Nuclear receptor binding protein
E0789344	Amniota	Metazoa	Transcription initiation factor tfiid subunit
E0789485	Amniota	Metazoa	Sphingosine kinase
E0789653	Amniota	Metazoa	Dynactin subunit
E0789683	Amniota	Metazoa	Growth arrest specific
E0789718	Amniota	Metazoa	Ragulator complex protein lamtor5
E0790104	Amniota	Metazoa	RING-type E3 ubiquitin transferase
E0790576	Amniota	Metazoa	Ligand of numb-protein
E0792967	Amniota	Opisthokonta	Rna polymerase
E0792988	Amniota	Opisthokonta	Diacylglycerol kinase
E0793126	Amniota	Opisthokonta	RNA helicase
E0793187	Amniota	Opisthokonta	Serine/threonine kinase
E0793370	Amniota	Opisthokonta	YEATS domain-containing protein
E0793441	Amniota	Opisthokonta	Transmembrane channel-like protein
E0793449	Amniota	Opisthokonta	Peroxisomal biogenesis factor 11
E0793477	Amniota	Opisthokonta	Helicase lymphoid specific
E0793928	Amniota	Opisthokonta	Dna damage-regulated autophagy modulator protein
E0794206	Amniota	Opisthokonta	Inflammation and lipid regulator with UBA-like and NBR1-like domains
E0795490	Amniota	Opisthokonta	Small ribosomal subunit protein bS6m
E0795765	Amniota	Opisthokonta	Protein xrp2
E0801530	Amniota	Eukaryota	Syntaxin binding protein
E0801542	Amniota	Eukaryota	Nucleoside diphosphate-linked moiety
E0801654	Amniota	Eukaryota	Dolichyl-diphosphooligosaccharide–protein glycotransferase
E0801796	Amniota	Eukaryota	Protein mak16 homolog
E0802095	Amniota	Eukaryota	Derived by automated computational analysis using gene prediction method Gnomon
E0802102	Amniota	Eukaryota	60S ribosome subunit biogenesis protein nip7
E0802214	Amniota	Eukaryota	Reductase
E0802469	Amniota	Eukaryota	Succinate dehydrogenase ubiquinone flavoprotein subunit mitochondrial
E0802500	Amniota	Eukaryota	Eukaryotic translation initiation factor
E0802531	Amniota	Eukaryota	Peptidyl-prolyl cis-trans isomerase
E0802850	Amniota	Eukaryota	Sodium/bile acid cotransporter
E0803542	Amniota	Eukaryota	Tetratricopeptide repeat
E0803734	Amniota	Eukaryota	Alpha-methylacyl-coa racemase
E0804547	Amniota	Eukaryota	Eukaryotic translation initiation factor 4C

Supplementary Table 1. (Continued.)

HOG ID	Benchmark set	Taxonomic level	Functional description
E0804746	Amniota	Eukaryota	AMMECR1 domain-containing protein
E0804997	Amniota	Eukaryota	Serine-threonine kinase receptor-associated protein
E0805071	Amniota	Eukaryota	TRAF3-interacting protein
E0805082	Amniota	Eukaryota	Acid phosphatase
E0805169	Amniota	Eukaryota	WD repeat containing antisense to TP73
E0805724	Amniota	Eukaryota	ATP synthase subunit
E0805956	Amniota	Eukaryota	Integrator complex subunit 14
E0808060	Amniota	Eukaryota	Dehydrogenase quinone
E0808147	Amniota	Eukaryota	Mannosyl-oligosaccharide glucosidase
E0809946	Amniota	Eukaryota	Leucine zipper transcription factor-like protein
E0810770	Amniota	Eukaryota	F-box protein
E0811408	Amniota	Eukaryota	Proteasome activator complex subunit
E0830763	Amniota	Eukaryota	NADH dehydrogenase ubiquinone
E1027385	Amniota	LUCA	Elongation factor
E1027398	Amniota	LUCA	Glycogen starch synthase
E1033126	Amniota	LUCA	Mannose-6-phosphate isomerase
E0722237	Primates	Simiiformes	Zinc finger protein 528
E0723174	Primates	Primates	neuropeptide
E0723659	Primates	Euarchontoglires	zinc finger protein 426
E0724651	Primates	Boreoeutheria	colipase like
E0724835	Primates	Boreoeutheria	Secreted protein
E0727291	Primates	Eutheria	fetal and adult testis expressed
E0727636	Primates	Eutheria	zinc finger protein 529
E0727690	Primates	Eutheria	Membrane spanning 4-domains A14
E0730033	Primates	Theria	neuropeptide FF-amide peptide precursor
E0730175	Primates	Theria	secreted LY6/PLAUR domain containing
E0730840	Primates	Theria	natural cytotoxicity triggering receptor
E0731014	Primates	Theria	Small integral membrane protein 12
E0731589	Primates	Mammalia	PABPN1 like cytoplasmic
E0731633	Primates	Mammalia	family with sequence similarity 104 member
E0731675	Primates	Mammalia	hemoglobin subunit mu
E0731702	Primates	Mammalia	linker for activation of
E0731827	Primates	Mammalia	Fc fragment of IgE receptor II
E0732264	Primates	Mammalia	LLLL and CFNLAS motif containing
E0732280	Primates	Mammalia	Serine rich single-pass membrane protein
E0732400	Primates	Mammalia	interleukin
E0732604	Primates	Amniota	spermatogenesis associated 46
E0732774	Primates	Amniota	ubiquitin specific peptidase 21
E0733555	Primates	Amniota	chromosome 19 open reading frame 38
E0733700	Primates	Amniota	zinc finger protein 12
E0735286	Primates	Amniota	interleukin-23 subunit alpha
E0736175	Primates	Amniota	prickle planar cell polarity protein
E0736543	Primates	Tetrapoda	natriuretic peptides
E0736748	Primates	Tetrapoda	BPI fold containing family
E0736831	Primates	Tetrapoda	cilia and flagella associated protein 126
E0737727	Primates	Sarcopterygii	–arginine ADP-ribosyltransferase
E0737776	Primates	Sarcopterygii	transcription factor 15
E0738081	Primates	Sarcopterygii	inka box actin regulator
E0738139	Primates	Sarcopterygii	BAF chromatin remodeling complex subunit BCL7C
E0738291	Primates	Sarcopterygii	brain expressed associated with Nedd4
E0738390	Primates	Sarcopterygii	microfibril associated protein
E0739110	Primates	Euteleostomi	zinc finger DHHC-type
E0739292	Primates	Euteleostomi	S100 calcium binding protein
E0739299	Primates	Euteleostomi	toll-like receptor
E0739722	Primates	Euteleostomi	synapse differentiation inducing
E0743439	Primates	Euteleostomi	Uncharacterized protein
E0745504	Primates	Gnathostomata	single-pass membrane protein with coiled-coil domains
E0745593	Primates	Gnathostomata	myozenin
E0745800	Primates	Gnathostomata	phosphoinositide interacting regulator of transient receptor potential channels
E0745803	Primates	Gnathostomata	phosphoinositide-3-kinase regulatory subunit
E0745882	Primates	Gnathostomata	transcription factor
E0746007	Primates	Gnathostomata	5-hydroxytryptamine receptor 1A
E0746136	Primates	Gnathostomata	prickle planar cell polarity protein
E0746397	Primates	Gnathostomata	epidermal growth factor receptor kinase substrate 8-like protein

Supplementary Table 1. (Continued.)

HOG ID	Benchmark set	Taxonomic level	Functional description
E0746720	Primates	Gnathostomata	family with sequence similarity 83 member
E0746976	Primates	Gnathostomata	transmembrane protein 150B
E0746990	Primates	Gnathostomata	profilin
E0747254	Primates	Gnathostomata	LY6/PLAUR domain containing 6B
E0747346	Primates	Gnathostomata	transmembrane protein 82
E0747746	Primates	Gnathostomata	proline rich
E0748908	Primates	Gnathostomata	receptor protein-tyrosine kinase
E0748910	Primates	Gnathostomata	cellular communication network factor
E0749141	Primates	Gnathostomata	H2.0 like homeobox
E0749156	Primates	Gnathostomata	complement C1s
E0752012	Primates	Gnathostomata	DPY30 domain containing
E0752621	Primates	Vertebrata	LY6/PLAUR domain containing
E0753969	Primates	Vertebrata	ST8 alpha-N-acetyl-neuraminide alpha-2,8- sialyltransferase
E0754037	Primates	Vertebrata	ferric chelate reductase
E0754187	Primates	Vertebrata	serum amyloid
E0756044	Primates	Chordata	solute carrier family
E0756517	Primates	Chordata	coiled-coil domain containing 183
E0758276	Primates	Chordata	Kringle-containing protein marking the eye and the nose
E0758872	Primates	Deuterostomia	oxidase subunit 7A2 mitochondrial
E0759150	Primates	Deuterostomia	glypican
E0759261	Primates	Deuterostomia	matrix AAA peptidase interacting protein
E0761286	Primates	Bilateria	tubulointerstitial nephritis antigen like
E0761287	Primates	Bilateria	tRNA selenocysteine 1-associated protein
E0761914	Primates	Bilateria	TGF-beta activated kinase
E0777912	Primates	Eumetazoa	M-phase phosphoprotein
E0778345	Primates	Eumetazoa	mediator of RNA polymerase ii transcription subunit
E0780321	Primates	Eumetazoa	transcription factor
E0781265	Primates	Eumetazoa	spermatogenesis associated
E0789356	Primates	Metazoa	factor interacting protein
E0789656	Primates	Metazoa	mediator of RNA polymerase ii transcription subunit 18
E0793630	Primates	Opisthokonta	RNA polymerase III transcription initiation factor
E0794206	Primates	Opisthokonta	inflammation and lipid regulator with UBA-like and NBR1-like domains
E0795703	Primates	Opisthokonta	selenoprotein
E0797424	Primates	Opisthokonta	TATA-box binding protein associated factor 15
E0801502	Primates	Eukaryota	isovaleryl-coa dehydrogenase mitochondrial
E0802195	Primates	Eukaryota	sigma non-opioid intracellular receptor
E0802263	Primates	Eukaryota	Peptidylprolyl isomerase domain and wd
E0802523	Primates	Eukaryota	RNA 3'-terminal phosphate cyclase-like protein
E0802587	Primates	Eukaryota	Derived by automated computational analysis using gene prediction method Gnomon
E0803033	Primates	Eukaryota	ERI1 exoribonuclease
E0804986	Primates	Eukaryota	protein tex261
E0805984	Primates	Eukaryota	patch domain-containing protein 11
E0807183	Primates	Eukaryota	SAP30 binding protein
E0808942	Primates	Eukaryota	SCP2 domain-containing protein
E0811210	Primates	Eukaryota	spermatogenesis associated
E0813529	Primates	Eukaryota	N-acetyltransferase domain-containing protein
E0827794	Primates	Eukaryota	Peptidase S1 domain-containing protein
E0827817	Primates	Eukaryota	guanylate cyclase soluble subunit
E0829605	Primates	Eukaryota	golgi-associated plant pathogenesis-related protein
E1027752	Primates	LUCA	Peptidase M20 dimerisation domain-containing protein
E1033369	Primates	LUCA	N-acyl-aromatic-L-amino acid amidohydrolase
E1033459	Primates	LUCA	transport and golgi organization
E0866364	Bacteria	Campylobacterales	Ribosomal RNA large subunit methyltransferase
E0866384	Bacteria	Campylobacterales	Putative uncharacterized protein
E0866386	Bacteria	Campylobacterales	Competence locus
E0866388	Bacteria	Campylobacterales	Putative uncharacterized protein
E0866440	Bacteria	Campylobacterales	Rare lipoprotein
E0866444	Bacteria	Campylobacterales	Cell division protein
E0866925	Bacteria	Campylobacteria	Putative uncharacterized protein
E0866981	Bacteria	Campylobacteria	Ligase
E0886776	Bacteria	Bacteroidia	Cell division protein
E0889623	Bacteria	Bacteroidota	D-isomer specific 2-hydroxyacid dehydrogenase
E0889653	Bacteria	Bacteroidota	Membrane protein involved in the export of O-antigen and teichoic acid

Supplementary Table 1. (Continued.)

HOG ID	Benchmark set	Taxonomic level	Functional description
E0889658	Bacteria	Bacteroidota	Repeat protein
E0983296	Bacteria	Gammaproteobacteria	Outer membrane
E0988205	Bacteria	Proteobacteria	Signal peptide peptidase SppA 36K type
E0990658	Bacteria	Bacteria	DNA methylase N-4/N-6 domain protein
E0990732	Bacteria	Bacteria	Probable queuosine precursor transporter
E0990851	Bacteria	Bacteria	Sec-independent protein translocase protein
E0990893	Bacteria	Bacteria	Acetyltransferase gnat family
E0990905	Bacteria	Bacteria	EscU/YscU/HrcU family type III secretion system export apparatus switch protein
E0990910	Bacteria	Bacteria	Ligase
E0990983	Bacteria	Bacteria	Thiamine biosynthesis protein this
E0991027	Bacteria	Bacteria	Protein of unknown function DUF395 YeeE/YedE
E0991055	Bacteria	Bacteria	B-type cytochrome subunit
E0991069	Bacteria	Bacteria	Hydrogenase nickel insertion protein hypa
E0991084	Bacteria	Bacteria	Hypothetical protein
E0991092	Bacteria	Bacteria	Electron transport protein SCO1/SenC
E0991113	Bacteria	Bacteria	Family protein
E0991463	Bacteria	Bacteria	Outer membrane protein
E0991547	Bacteria	Bacteria	C-5 cytosine-specific DNA methylase
E0991584	Bacteria	Bacteria	Caax amino terminal protease family
E0992157	Bacteria	Bacteria	Creatininase
E0992254	Bacteria	Bacteria	Crispr-associated protein cas2
E0992482	Bacteria	Bacteria	Putative uncharacterized protein
E0992697	Bacteria	Bacteria	Cobalamin biosynthesis protein
E0993605	Bacteria	Bacteria	Type IV secretory pathway VirB4
E0994123	Bacteria	Bacteria	Cobalamin vitamin B12 biosynthesis CbiG protein
E0994427	Bacteria	Bacteria	Alkaline phosphatase
E0994512	Bacteria	Bacteria	Tap domain protein
E0994775	Bacteria	Bacteria	Phytanoyl-CoA dioxygenase
E0995117	Bacteria	Bacteria	Protein of unknown function DUF980
E0995137	Bacteria	Bacteria	Nitrogenase iron protein
E0995409	Bacteria	Bacteria	Small basic protein
E0995484	Bacteria	Bacteria	PHP domain protein
E0995820	Bacteria	Bacteria	DTW domain containing protein
E0996090	Bacteria	Bacteria	RNA polymerase sigma-24 subunit ECF subfamily
E0996091	Bacteria	Bacteria	Taurine dioxygenase
E0997625	Bacteria	Bacteria	Glycoside hydrolase family 43
E0998388	Bacteria	Bacteria	Protein of unknown function DUF839
E1000672	Bacteria	Bacteria	Protein of unknown function DUF3109
E1001108	Bacteria	Bacteria	Sec-c motif domain protein
E1001680	Bacteria	Bacteria	MazG nucleotide pyrophosphohydrolase
E1001738	Bacteria	Bacteria	Glycoside hydrolase family 25
E1002243	Bacteria	Bacteria	Glycoside hydrolase family 43
E1008850	Bacteria	Bacteria	Phosphate acetyltransferase
E1013559	Bacteria	Bacteria	Oxidoreductase cytochrome c1
E1026438	Bacteria	Bacteria	Lyase
E1027257	Bacteria	LUCA	Cell division protein ftsz
E1027266	Bacteria	LUCA	Large ribosomal subunit protein uL2
E1027329	Bacteria	LUCA	Thymidine phosphorylase
E1027338	Bacteria	LUCA	Methylthioribose-1-phosphate isomerase
E1027387	Bacteria	LUCA	Small ribosomal subunit protein uS10
E1027550	Bacteria	LUCA	Ribosomal silencing factor RsfS
E1027632	Bacteria	LUCA	Gpr1/fun34/yaah family protein
E1027911	Bacteria	LUCA	Conserved hypothetical protein
E1027973	Bacteria	LUCA	Dna mismatch repair protein mutl
E1028216	Bacteria	LUCA	Porphobilinogen deaminase
E1028269	Bacteria	LUCA	Polyphosphate kinase
E1028473	Bacteria	LUCA	Queuine trna-ribosyltransferase
E1028477	Bacteria	LUCA	Epoxyqueuosine reductase QueH
E1028560	Bacteria	LUCA	Fructose-1,6-bisphosphatase class ii
E1029002	Bacteria	LUCA	Fumarate hydratase class
E1029250	Bacteria	LUCA	Potassium transport system protein kup
E1029441	Bacteria	LUCA	Ethanolamine ammonia-lyase large subunit
E1029756	Bacteria	LUCA	Phosphoribosyl-amp cyclohydrolase

Supplementary Table 1. (Continued.)

HOG ID	Benchmark set	Taxonomic level	Functional description
E1029773	Bacteria	LUCA	Protein of unknown function DUF89
E1030072	Bacteria	LUCA	Redox-active disulfide protein
E1030201	Bacteria	LUCA	Deoxynucleoside kinase
E1030314	Bacteria	LUCA	Molybdopterin converting factor
E1030603	Bacteria	LUCA	Methyltransferase
E1030655	Bacteria	LUCA	Alpha alpha-trehalose-phosphate synthase
E1030751	Bacteria	LUCA	Ribonuclease iii
E1030960	Bacteria	LUCA	Ferrochelatase
E1033107	Bacteria	LUCA	Lipid-a-disaccharide synthase
E1033225	Bacteria	LUCA	30s ribosomal protein s9
E1033231	Bacteria	LUCA	Ribosomal protein s8
E1033232	Bacteria	LUCA	30s ribosomal protein s17
E1033238	Bacteria	LUCA	30s ribosomal protein s12
E1033252	Bacteria	LUCA	Isocitrate dehydrogenase
E1033823	Bacteria	LUCA	PSP1 domain protein
E1033950	Bacteria	LUCA	Phenylalanyl-trna synthetase alpha
E1033955	Bacteria	LUCA	N- 5'-phosphoribosyl anthranilate isomerase
E1033964	Bacteria	LUCA	Ribosomal RNA large subunit methyltransferase
E1036044	Bacteria	LUCA	Glutathionylspermidine synthase
E1036190	Bacteria	LUCA	Ribosomal protein l7/l12
E1036237	Bacteria	LUCA	Formate dehydrogenase gamma subunit
E1036285	Bacteria	LUCA	Protein of unknown function DUF808
E1036895	Bacteria	LUCA	Transferase
E1037234	Bacteria	LUCA	DUF2238 domain-containing protein
E1038404	Bacteria	LUCA	Transcription termination/antitermination protein nusA
E1039751	Bacteria	LUCA	Mg chelatase subunit chli
E0906941	Actinomycetes	Mycobacterium	Exported alanine and valine rich protein
E0908369	Actinomycetes	Mycobacteriaceae	Conserved membrane protein of uncharacterized function
E0908385	Actinomycetes	Mycobacteriaceae	Hypothetical protein
E0908420	Actinomycetes	Mycobacteriaceae	Hypothetical protein
E0908431	Actinomycetes	Mycobacteriaceae	Transcriptional regulator
E0908698	Actinomycetes	Mycobacteriaceae	PE family protein
E0908753	Actinomycetes	Mycobacteriaceae	Substrate-binding region of ABC-type glycine betaine transport system
E0908869	Actinomycetes	Mycobacteriaceae	Hypothetical protein
E0909355	Actinomycetes	Mycobacteriales	Transcriptional regulator LysR family
E0909399	Actinomycetes	Mycobacteriales	Putative uncharacterized protein
E0909416	Actinomycetes	Mycobacteriales	Two component transcriptional regulator LuxR family
E0909556	Actinomycetes	Mycobacteriales	Membrane protein
E0909967	Actinomycetes	Mycobacteriales	Acyltransferase
E0910650	Actinomycetes	Mycobacteriales	Hypothetical protein
E0910862	Actinomycetes	Actinomycetia	Putative uncharacterized protein
E0910923	Actinomycetes	Actinomycetia	Putative uncharacterized protein
E0910958	Actinomycetes	Actinomycetia	Major facilitator superfamily
E0910963	Actinomycetes	Actinomycetia	Putative uncharacterized protein
E0910969	Actinomycetes	Actinomycetia	Molybdopterin converting factor
E0911000	Actinomycetes	Actinomycetia	Proton-translocating NADH-quinone oxidoreductase chain
E0911214	Actinomycetes	Actinomycetia	Helix-turn-helix domain protein
E0911256	Actinomycetes	Actinomycetia	Malonyl CoA-acyl carrier protein transacylase FabD2
E0911917	Actinomycetes	Actinomycetia	Putative uncharacterized protein
E0911951	Actinomycetes	Actinomycetia	Membrane protein
E0912050	Actinomycetes	Actinomycetia	Putative uncharacterized protein
E0912307	Actinomycetes	Actinomycetia	DNA polymerase III
E0912826	Actinomycetes	Actinomycetia	Roadblock/LC7 family protein
E0913273	Actinomycetes	Actinomycetia	Oxidoreductase molybdopterin binding
E0913327	Actinomycetes	Actinomycetia	ABC transporter
E0913950	Actinomycetes	Actinomycetia	Transcriptional regulator
E0913985	Actinomycetes	Actinomycetia	Transcriptional regulatory protein
E0914187	Actinomycetes	Actinomycetia	Hypothetical protein
E0914813	Actinomycetes	Actinomycetia	Hypothetical protein
E0915110	Actinomycetes	Actinomycetia	Polyketide synthase associated protein
E0916679	Actinomycetes	Actinobacteriota	ADP-ribosylation/Crystallin J1
E0916784	Actinomycetes	Actinobacteriota	Hypothetical protein
E0916882	Actinomycetes	Actinobacteriota	Signal transduction histidine kinase
E0916891	Actinomycetes	Actinobacteriota	PF07905 purine catabolism regulatory protein-like family

Supplementary Table 1. (Continued.)

HOG ID	Benchmark set	Taxonomic level	Functional description
E0916905	Actinomycetes	Actinobacteriota	Membrane protein
E0917144	Actinomycetes	Actinobacteriota	Putative uncharacterized protein
E0990835	Actinomycetes	Bacteria	Ion transport protein
E0990879	Actinomycetes	Bacteria	Rod shape-determining protein mreC
E0990904	Actinomycetes	Bacteria	Ferredoxin oxidoreductase
E0991133	Actinomycetes	Bacteria	Hydrogenase maturation protease
E0991409	Actinomycetes	Bacteria	TRNA Ile -lysidine
E0991460	Actinomycetes	Bacteria	Dihydroneopterin aldolase
E0992129	Actinomycetes	Bacteria	Endonuclease
E0992136	Actinomycetes	Bacteria	5,10-methylenetetrahydrofolate reductase
E0992179	Actinomycetes	Bacteria	Glutamate synthase
E0992182	Actinomycetes	Bacteria	Phosphodiesterase mj0936 family
E0992194	Actinomycetes	Bacteria	Putative uncharacterized protein
E0992444	Actinomycetes	Bacteria	Putative uncharacterized protein
E0992711	Actinomycetes	Bacteria	Hypothetical protein
E0992735	Actinomycetes	Bacteria	NADH pyrophosphatase
E0993664	Actinomycetes	Bacteria	N-succinyltransferase
E0994533	Actinomycetes	Bacteria	MmcQ/YjbR family DNA-binding protein
E0995457	Actinomycetes	Bacteria	Putative uncharacterized protein
E0995474	Actinomycetes	Bacteria	Thiamin pyrophosphokinase catalytic region
E0996733	Actinomycetes	Bacteria	Iron permease
E0998291	Actinomycetes	Bacteria	Gas vesicle
E0998809	Actinomycetes	Bacteria	Hypothetical protein
E1000494	Actinomycetes	Bacteria	Putative transcriptional regulator Crp/Fnr family
E1001514	Actinomycetes	Bacteria	Proteasome subunit beta
E1005383	Actinomycetes	Bacteria	Nuclear transport factor
E1006095	Actinomycetes	Bacteria	Hypothetical protein
E1007742	Actinomycetes	Bacteria	FHA domain containing protein
E1008293	Actinomycetes	Bacteria	Integration host factor
E1009211	Actinomycetes	Bacteria	Putative uncharacterized protein
E1015623	Actinomycetes	Bacteria	Hypothetical protein
E1016676	Actinomycetes	Bacteria	Serine protease
E1025643	Actinomycetes	Bacteria	N-acetylmuramoyl-L-alanine amidase
E1027257	Actinomycetes	LUCA	Cell division protein ftsz
E1027299	Actinomycetes	LUCA	Glycine–tRNA ligase
E1027489	Actinomycetes	LUCA	3-octaprenyl-4-hydroxybenzoate carboxy-lyase
E1027651	Actinomycetes	LUCA	Phosphatidylserine decarboxylase
E1027665	Actinomycetes	LUCA	Quinolinate synthetase
E1027669	Actinomycetes	LUCA	L-lactate permease
E1027736	Actinomycetes	LUCA	Mannose-1-phosphate guanylyltransferase
E1028017	Actinomycetes	LUCA	Osmotically inducible protein
E1028175	Actinomycetes	LUCA	Hydantoin racemase
E1028192	Actinomycetes	LUCA	UPF0060 membrane protein
E1028268	Actinomycetes	LUCA	Putative uncharacterized protein
E1028900	Actinomycetes	LUCA	Glutamate racemase
E1029110	Actinomycetes	LUCA	Holliday junction dna helicase ruva
E1029913	Actinomycetes	LUCA	Trna delta
E1029981	Actinomycetes	LUCA	Trna pseudouridine synthase
E1030506	Actinomycetes	LUCA	Gcn5-related N-acetyltransferase
E1031052	Actinomycetes	LUCA	Sulfate abc transporter
E1031550	Actinomycetes	LUCA	Hypothetical protein
E1032154	Actinomycetes	LUCA	Linocin_M18 bacteriocin protein
E1032317	Actinomycetes	LUCA	Hypothetical protein
E1032889	Actinomycetes	LUCA	Spoom family protein
E1033234	Actinomycetes	LUCA	50s ribosomal protein l19
E1033765	Actinomycetes	LUCA	Domain-containing protein
E1035591	Actinomycetes	LUCA	Transcriptional regulator ArsR family
E1036199	Actinomycetes	LUCA	Protein of unknown function DUF179
E1037740	Actinomycetes	LUCA	Hypothetical protein
E1039273	Actinomycetes	LUCA	Branched-chain amino acid transport system ii carrier protein
E1039842	Actinomycetes	LUCA	DNA topoisomerase
E1040100	Actinomycetes	LUCA	YiaAB two helix domain protein
E0758622	Eukaryota large	Chordata	cadherin
E0765773	Eukaryota large	Bilateria	neurexophilin and pc-esterase domain family member

Supplementary Table 1. (Continued.)

HOG ID	Benchmark set	Taxonomic level	Functional description
E0778026	Eukaryota large	Eumetazoa	Derived by automated computational analysis using gene prediction method Gnomon
E0789365	Eukaryota large	Metazoa	glutamate receptor
E0792959	Eukaryota large	Opisthokonta	t-SNARE coiled-coil homology domain-containing protein
E0792966	Eukaryota large	Opisthokonta	splicing factor U2AF subunit
E0792972	Eukaryota large	Opisthokonta	ectonucleoside triphosphate diphosphohydrolase
E0792981	Eukaryota large	Opisthokonta	fatty acid hydroxylase domain-containing protein
E0792984	Eukaryota large	Opisthokonta	u1 small nuclear ribonucleoprotein 70 kDa
E0793012	Eukaryota large	Opisthokonta	ras-related protein
E0793013	Eukaryota large	Opisthokonta	tyrosine-protein kinase
E0793020	Eukaryota large	Opisthokonta	thioredoxin
E0793095	Eukaryota large	Opisthokonta	5'-nucleotidase domain containing
E0793129	Eukaryota large	Opisthokonta	delta 3,5 -Delta 2,4 -dienoyl-CoA isomerase
E0793140	Eukaryota large	Opisthokonta	sorting
E0793181	Eukaryota large	Opisthokonta	transmembrane emp24 domain-containing protein
E0793207	Eukaryota large	Opisthokonta	ankyrin repeat
E0793209	Eukaryota large	Opisthokonta	solute carrier family 26 member
E0793266	Eukaryota large	Opisthokonta	polypyrimidine tract binding protein
E0793469	Eukaryota large	Opisthokonta	carboxylic ester hydrolase
E0793516	Eukaryota large	Opisthokonta	major facilitator superfamily
E0801481	Eukaryota large	Eukaryota	regulator of chromosome condensation
E0801602	Eukaryota large	Eukaryota	Amino acid transporter transmembrane domain-containing protein
E0801607	Eukaryota large	Eukaryota	Serine aminopeptidase S33 domain-containing protein
E0801729	Eukaryota large	Eukaryota	charged multivesicular body protein
E0801760	Eukaryota large	Eukaryota	autophagy-related protein
E0801764	Eukaryota large	Eukaryota	mitochondrial-processing peptidase subunit
E0801815	Eukaryota large	Eukaryota	phosphoglycerate mutase
E0801822	Eukaryota large	Eukaryota	casein kinase ii subunit alpha
E0801833	Eukaryota large	Eukaryota	ornithine decarboxylase
E0801848	Eukaryota large	Eukaryota	nad-dependent protein
E0801856	Eukaryota large	Eukaryota	U6 snRNA-associated sm-like protein lsm6
E0801868	Eukaryota large	Eukaryota	choline transporter-like protein
E0801870	Eukaryota large	Eukaryota	Protein N-terminal and lysine N-methyltransferase efm7
E0801887	Eukaryota large	Eukaryota	replication factor
E0801902	Eukaryota large	Eukaryota	Cyclin
E0801907	Eukaryota large	Eukaryota	Large ribosomal subunit protein uL23
E0801918	Eukaryota large	Eukaryota	Derived by automated computational analysis using gene prediction method Gnomon
E0801941	Eukaryota large	Eukaryota	RBR-type E3 ubiquitin transferase
E0801945	Eukaryota large	Eukaryota	NADH-cytochrome b5 reductase
E0801960	Eukaryota large	Eukaryota	cornichon family AMPA receptor auxiliary protein
E0801973	Eukaryota large	Eukaryota	peptidylprolyl isomerase
E0802012	Eukaryota large	Eukaryota	serine palmitoyltransferase long chain base subunit
E0802046	Eukaryota large	Eukaryota	ethanolamine kinase
E0802055	Eukaryota large	Eukaryota	Derived by automated computational analysis using gene prediction method Gnomon
E0802134	Eukaryota large	Eukaryota	AAA+ ATPase domain-containing protein
E0802137	Eukaryota large	Eukaryota	rna helicase
E0802203	Eukaryota large	Eukaryota	cystathionine
E0802221	Eukaryota large	Eukaryota	cell cycle control protein
E0802233	Eukaryota large	Eukaryota	serine incorporator
E0802298	Eukaryota large	Eukaryota	charged multivesicular body protein
E0802319	Eukaryota large	Eukaryota	defective in cullin neddylation
E0802323	Eukaryota large	Eukaryota	Magnesium transporter
E0802328	Eukaryota large	Eukaryota	serine/threonine-protein phosphatase 2a 55 kda regulatory subunit
E0802335	Eukaryota large	Eukaryota	equilibrative nucleoside transporter
E0802337	Eukaryota large	Eukaryota	Ubiquitin-like domain-containing protein
E0802395	Eukaryota large	Eukaryota	casein kinase ii subunit beta
E0802409	Eukaryota large	Eukaryota	Sodium/calcium exchanger membrane region domain-containing protein
E0802418	Eukaryota large	Eukaryota	vesicle-associated membrane protein
E0802421	Eukaryota large	Eukaryota	V-type proton ATPase subunit
E0802457	Eukaryota large	Eukaryota	peroxisomal membrane protein
E0802506	Eukaryota large	Eukaryota	60s ribosomal protein l21
E0802572	Eukaryota large	Eukaryota	ribonucleoside-diphosphate reductase subunit m2

Supplementary Table 1. (Continued.)

HOG ID	Benchmark set	Taxonomic level	Functional description
E0802609	Eukaryota large	Eukaryota	pyruvate decarboxylase
E0802642	Eukaryota large	Eukaryota	t-SNARE coiled-coil homology domain-containing protein
E0802656	Eukaryota large	Eukaryota	carboxylic ester hydrolase
E0802701	Eukaryota large	Eukaryota	oxidase
E0802750	Eukaryota large	Eukaryota	Derived by automated computational analysis using gene prediction method Gnomon
E0802759	Eukaryota large	Eukaryota	TatD DNase domain containing
E0802823	Eukaryota large	Eukaryota	ABC1 atypical kinase-like domain-containing protein
E0802949	Eukaryota large	Eukaryota	amine oxidase
E0802977	Eukaryota large	Eukaryota	Derived by automated computational analysis using gene prediction method Gnomon
E0803038	Eukaryota large	Eukaryota	cysteine and glycine-rich protein
E0803087	Eukaryota large	Eukaryota	Amidase domain-containing protein
E0803398	Eukaryota large	Eukaryota	CSC1/OSCA1-like 7TM region domain-containing protein
E0803403	Eukaryota large	Eukaryota	Derived by automated computational analysis using gene prediction method Gnomon
E0803479	Eukaryota large	Eukaryota	RING-type E3 ubiquitin transferase
E0804281	Eukaryota large	Eukaryota	protein-serine/threonine phosphatase
E0804434	Eukaryota large	Eukaryota	Serine/threonine protein phosphatase 2a regulatory subunit
E0804483	Eukaryota large	Eukaryota	C2H2-type domain-containing protein
E0804978	Eukaryota large	Eukaryota	protein yippee-like
E0805099	Eukaryota large	Eukaryota	Bidirectional sugar transporter sweet
E0805279	Eukaryota large	Eukaryota	phosphotransferase
E0805484	Eukaryota large	Eukaryota	Derived by automated computational analysis using gene prediction method Gnomon
E0805752	Eukaryota large	Eukaryota	domain-containing protein
E0805789	Eukaryota large	Eukaryota	chloride intracellular channel protein
E0805822	Eukaryota large	Eukaryota	voltage-dependent anion-selective channel protein
E0806548	Eukaryota large	Eukaryota	domain-containing protein
E0809154	Eukaryota large	Eukaryota	UDP-glucuronosyltransferase
E0810427	Eukaryota large	Eukaryota	chitinase
E0811656	Eukaryota large	Eukaryota	annexin
E1027260	Eukaryota large	LUCA	ribose-phosphate pyrophosphokinase
E1027275	Eukaryota large	LUCA	Small ribosomal subunit protein uS5
E1027281	Eukaryota large	LUCA	proteasome subunit alpha type
E1027363	Eukaryota large	LUCA	Developmentally regulated gtp binding protein
E1027592	Eukaryota large	LUCA	L-lactate dehydrogenase
E1027833	Eukaryota large	LUCA	Enoyl reductase ER domain-containing protein
E1028318	Eukaryota large	LUCA	solute carrier family 23 member
E1028583	Eukaryota large	LUCA	FMN hydroxy acid dehydrogenase domain-containing protein
E1028629	Eukaryota large	LUCA	ammonium transporter rh type

Supplementary Table 2. List of sequence aligners and command lines used to run the benchmark. Each aligner name corresponds to the legend in Fig. 2a. Options used to set the number of CPU threads are omitted.

Aligner name	Version	Command line
ClustalΩ	v1.2.4	`clustalo -i <input.fa>`
ClustalW	v2.1	`clustalw2 -INFILE=<input.fa> -QUIET -OUTPUT=FASTA -TYPE=PROTEIN -OUTFILE=<output.fa> &>/dev/null`
FAMSA	v2.2.3	`famsa <input.fa> STDOUT 2>/dev/null`
MAFFT v7	v7.526	`mafft --anysymbol --quiet <input.fa>`
MAFFT v6	v6.864	`mafft --anysymbol --quiet <input.fa>`
L-INS-i v7	v7.526	`mafft-linsi --anysymbol --quiet <input.fa>`
MUSCLE	v5.3	`muscle -align <input.fa> -output /dev/stdout 2>/dev/null`

Supplementary Table 3. Evolutionary model selected before and after enriching Amniota HOGs. Each HOG ID can be accessed in the OMA browser by querying `HOG:<HOG ID>`. Model (before/after) indicates the evolutionary model chosen by IQ-TREE ModelFinder before and after enriching Amniota HOGs (corresponding to Supplementary Fig. 3g–i).

HOG ID	Model (before)	Model (after)
E0732656	Q.bird+I	Q.bird+I+G4
E0736903	Q.mammal+I+G4	JTT+F+R4
E0738993	Q.plant+G4	Q.pfam+F+G4
E0739117	Q.bird+G4	WAG+I+G4
E0739356	Q.bird+F+G4	Q.insect+F+I+G4
E0739363	Q.mammal+G4	Q.pfam+G4
E0740076	Q.mammal+G4	Q.insect+F+I+G4
E0740208	Q.mammal+G4	Q.plant+I+G4
E0745588	Q.bird+G4	LG+F+R5
E0745662	Q.insect	Q.plant+I+G4
E0745759	Q.bird+G4	Q.plant+I+G4
E0746040	Q.plant+G4	Q.plant+G4
E0746607	Q.bird+G4	Q.plant+R5
E0746839	Q.bird+G4	Q.pfam+I+R4
E0747039	Q.bird+G4	Q.plant+I+G4
E0747049	Q.bird+G4	Q.mammal+G4
E0748381	Q.mammal+F+G4	Q.mammal+F+R3
E0749003	Q.bird+F+I	JTT+F+R4
E0749052	JTT+G4	Q.insect+F+I+G4
E0750288	Q.bird+F+G4	JTT+F+I+G4
E0752398	Q.bird+G4	mtInv+F+I+R5
E0752660	JTT+F+G4	Q.insect+F+I+G4
E0752678	JTT+G4	JTTDCMut+R6
E0754068	Q.mammal+G4	Q.mammal+I+G4
E0754499	Q.bird+G4	Q.pfam+I+G4
E0754546	Q.insect+F+G4	Q.insect+F+I+G4
E0755467	JTTDCMut+G4	Q.insect+F+G4
E0755569	JTT+G4	Q.pfam+R8
E0755637	Q.bird+G4	WAG+F+I+R4
E0756159	Q.mammal+G4	Q.pfam+R7
E0756270	Q.insect+G4	Q.yeast+F+G4
E0756421	Q.plant+I+G4	Q.pfam+R5
E0759640	FLU+F+G4	JTT+R4
E0760031	Q.mammal+I	JTT+R3
E0761363	JTT+G4	Q.insect+G4
E0761506	JTTDCMut+I	WAG+R4
E0761586	Q.bird+G4	Q.insect+G4
E0762353	Q.mammal+I+G4	Q.pfam+R7
E0771775	JTTDCMut+G4	LG+I+R7
E0778191	Q.mammal+G4	Q.insect+F+G4
E0778742	Q.insect+G4	Q.insect+R5
E0779849	Q.mammal+G4	LG+I+G4
E0779919	Q.mammal+R2	Q.pfam+R4
E0780141	Q.mammal+I+R2	Q.insect+F+R6
E0780552	Q.mammal+G4	Q.insect+I+G4
E0781053	JTT+G4	Q.insect+R5
E0781454	Q.bird+G4	Q.pfam+F+R5
E0783327	Q.mammal+G4	Q.mammal+R4
E0784079	Q.mammal+G4	JTT+R4
E0789041	Q.bird+I	Q.insect+R4
E0789139	Q.mammal+G4	Q.insect+R5
E0789344	Q.bird+I+G4	Q.insect+R5
E0789485	Q.mammal+G4	Q.pfam+F+I+R4
E0789653	Q.mammal+G4	Q.insect+G4
E0789683	JTTDCMut+R2	Q.pfam+I+G4
E0789718	Q.mammal+R2	Q.insect+G4
E0790104	Q.bird+G4	Q.pfam+R6
E0790576	Q.mammal+G4	JTTDCMut+F+R6
E0792967	Q.mammal+G4	Q.pfam+R4
E0792988	JTT+G4	Q.insect+R7
E0793126	JTT+R2	Q.pfam+I+R7
E0793187	Q.mammal+I+G4	Q.insect+R7

Supplementary Table 3. (Continued.)

HOG ID	Model (before)	Model (after)
E0793370	LG	LG+G4
E0793441	Q.mammal+G4	Q.pfam+R8
E0793449	Q.mammal+G4	Q.pfam+F+R4
E0793477	Q.plant+G4	LG+I+R6
E0793928	Q.plant+G4	LG+F+R5
E0794206	VT	Q.pfam+G4
E0795490	JTT+G4	LG+G4
E0795765	Q.bird+G4	LG+I+G4
E0801530	Q.insect+G4	Q.insect+R6
E0801542	JTT+G4	Q.pfam+I+G4
E0801654	JTT+I+R2	Q.pfam+R6
E0801796	Q.insect+R2	Q.insect+I+G4
E0802095	JTT+F+G4	Q.pfam+F+R7
E0802102	Q.bird	Q.insect+G4
E0802214	JTTDCMut+G4	LG+R7
E0802469	JTT+I+R2	LG+I+R5
E0802500	JTT+G4	Q.yeast+G4
E0802531	JTTDCMut+G4	Q.pfam+R5
E0802850	Q.plant+G4	LG+F+G4
E0803542	JTT+G4	Q.insect+R8
E0803734	Q.plant+G4	Q.pfam+R5
E0804547	JTT+G4	Q.insect+G4
E0804746	Q.insect+F+G4	Q.pfam+I+G4
E0804997	JTT+G4	Q.pfam+I+G4
E0805071	Q.mammal+F+G4	Q.insect+F+R4
E0805082	JTT+G4	Q.pfam+I+R5
E0805169	Q.mammal+G4	Q.pfam+F+G4
E0805724	mtVer+G4	mtZOA+R4
E0805956	Q.bird+I	LG+F+R5
E0808060	JTT+I+G4	Q.pfam+R5
E0808147	Q.plant+F+G4	Q.pfam+I+R5
E0809946	Q.mammal+G4	Q.insect+F+R4
E0810770	Q.mammal+G4	Q.pfam+F+R4
E0811408	Q.bird+G4	Q.insect+R5
E0830763	JTT+I	Q.pfam+I+G4
E1027385	JTT+I	LG+I+G4
E1027398	JTT+I+R2	LG+R5
E1033126	Q.bird+G4	Q.pfam+I+R5

Supplementary Table 4. Taxonomic impact on the gene trees by enrichment of Eukaryota HOGs. Each HOG ID can be accessed in the OMA browser by querying `HOG:<HOG ID>`. Entries are categorized based on their tree being improved (i.e. higher TCS scored) after enrichment. *Homologs within species* indicate the added number of sequences added with equal taxonomic origin to one of the input species, as a percentage to the number of original sequences. *Homologs outside species* quantifies the addition outside the input species boundary. Statistical summary ($\mu \pm SE$) of each category is presented at the end of the table.

HOG ID	Tree improved?	Homologs within species (%)	Homologs outside species (%)
E0737227	No	0	500
E0737958	No	0	500
E0739069	No	6.66667	493.333
E0739309	No	9.09091	490.909
E0741827	No	18.1818	481.818
E0746267	No	0	500
E0746710	No	0	500
E0754197	No	0	500
E0780419	No	0	500
E0781413	No	4.7619	495.238
E0792964	No	8.82353	491.176
E0793757	No	3.84615	496.154
E0794232	No	4.34783	495.652
E0794379	No	0	500
E0795420	No	5.55556	494.444
E0801614	No	2.38095	497.619
E0801829	No	0	500
E0803212	No	0	500
E0803328	No	5.26316	494.737
E0803575	No	13.3333	486.667
E0804898	No	0	500
E0805604	No	9.09091	490.909
E0808137	No	4.16667	495.833
E0736661	Yes	9.09091	490.909
E0736928	Yes	0	500
E0738913	Yes	0	500
E0738995	Yes	18.75	481.25
E0739275	Yes	0	500
E0739422	Yes	0	500
E0739485	Yes	6.66667	493.333
E0739504	Yes	0	500
E0739554	Yes	7.69231	492.308
E0739875	Yes	20	480
E0745703	Yes	5.26316	494.737
E0745717	Yes	0	500
E0745736	Yes	0	500
E0745773	Yes	0	500
E0745867	Yes	0	500
E0745959	Yes	0	500
E0745969	Yes	11.1111	488.889
E0746286	Yes	0	500
E0746357	Yes	0	500
E0746423	Yes	0	500
E0746523	Yes	0	500
E0746543	Yes	0	500
E0746659	Yes	0	500
E0747054	Yes	0	500
E0747393	Yes	0	500
E0748047	Yes	0	500
E0748254	Yes	0	500
E0749133	Yes	0	500
E0749136	Yes	11.7647	488.235
E0749142	Yes	7.14286	492.857
E0749861	Yes	7.14286	492.857
E0752475	Yes	5.88235	494.118
E0752720	Yes	0	500
E0753547	Yes	0	500
E0754233	Yes	0	500
E0756359	Yes	0	500

Supplementary Table 4. (Continued.)

HOG ID	Tree improved?	Homologs within species (%)	Homologs outside species (%)
E0758308	Yes	3.44828	496.552
E0758684	Yes	0	500
E0759084	Yes	5.55556	494.444
E0759259	Yes	4.16667	495.833
E0759639	Yes	0	500
E0761639	Yes	15.7895	484.211
E0769246	Yes	0	500
E0770269	Yes	3.0303	496.97
E0778401	Yes	0	500
E0779894	Yes	3.84615	496.154
E0780058	Yes	5.88235	494.118
E0781675	Yes	4.54545	495.455
E0781703	Yes	12.5	487.5
E0781792	Yes	0	500
E0783173	Yes	0	500
E0784066	Yes	7.40741	492.593
E0784079	Yes	7.40741	492.593
E0785287	Yes	43.75	456.25
E0789185	Yes	20	480
E0789260	Yes	13.0435	486.957
E0790046	Yes	0	500
E0790087	Yes	0	500
E0790149	Yes	0	500
E0793021	Yes	4.7619	495.238
E0793921	Yes	0	500
E0794199	Yes	3.44828	496.552
E0795316	Yes	4.16667	495.833
E0796129	Yes	0	500
E0801674	Yes	2	498
E0802240	Yes	0	500
E0802765	Yes	2.08333	497.917
E0803072	Yes	8.82353	491.176
E0803107	Yes	4.54545	495.455
E0803937	Yes	2.77778	497.222
E0804722	Yes	0	500
E0804798	Yes	0	500
E0812781	Yes	7.40741	492.593
E0813567	Yes	0	500
E0816287	Yes	0	500
E0816677	Yes	3.84615	496.154
E0818004	Yes	3.7037	496.296
**Summary**	No	4.15258 ± 1.03047	495.847 ± 1.03047
**Summary**	Yes	4.00576 ± 0.775259	495.994 ± 0.775257
